# Honey-Fuggling the Doctors

**Published:** 1899-07

**Authors:** 


### TEXAS MEDICAL JOURNAL.

AUSTIN, TEXAS.

A MONTHLY JOURNAL OF MEDICINE AND SURGERY.

F. E. DANIEL, M. D., Editor.

Published Monthly at Austin, Texas, by Drs. Daniel and Hudson. Subscription price $1.00 a year in advance.

Eastern Representative: John Guy Monihan, St. Paul Building, 220 Broadway, New York City.

Official organ of the West Texas Medical Association, the Houston District Medical Association, the Austin District Medical Society, the Brazos Valley Medical Association, the Galveston County Medical Society, and several others.

### Honey-Fuggling the Doctors.

In Texas the medical profession have no rights that a governor or a legislature feels bound (or inclined) to respect. Candidates cajole the doctors and their friends with sweet words and promises before election and then ignore them. The wishes of this large class of intelligent voters and their views as to reform in our sanitary and so-called "medical" laws, which views, it was given out, the governor was desirous of knowing, that he might intelligently state to the legislature what amendments should be made in them, were, to all appearances, entirely ignored. If ever he said a word on the subject it was never printed. The memorial from the State Medical Association sent in later elicited not the slightest attention so far as we are aware. "Told you so."

The Texas State Medical Association through a committee of its ablest and most experienced members, a committee of which the president, Dr. Wilson, was chairman, presented a bill to govern the practice of medicine in this State, a bill drawn up carefully after examining the laws of other states and getting the views of the members of the profession throughout the State, and this bill was put into the hands of a committee who, it seems, thought themselves capable of drawing up a better one. They suppressed the bill and substituted one of their own manufacture, a thing so entirely idiotic that the State Medical Association by resolution protested against its passage. The protest however is not what killed it. The determination to ignore the medical profession did it; the determination to not do anything for its members to protect either them or the blessed people from the human vultures (like that fellow at Fort Worth, for instance, with his "Husa" mixture of morphine which he was selling to the unfortunate gullibles as a "cure for morphinism.") The medical profession are not "in it," except as voters. How many more times are the doctors going to be made to believe that a small lightning bug is a political aurora-borealis? How much longer are they going to be deceived and gulled by promises?

We have said they have no rights which governors and legislatures feel oound to respect. It appears ( on paper) that there is reciprocity between the governing and the governed, in all good governments. Every citizen is taxed to carry on government. He must serve on juries; must do, or cause to be done, road work, etc. For this he is protected by law in his person, family, rights and property. The doctor moreover, is expected, if not required, to do much charity practice, and he does it, cheerfully. He can be attached and carried, by force, if necessary, to a distant point and there be made to give expert testimony,—give his opinion, in a case he knows nothing of as a *witness,* and at his own expense, and without compensation, or compensation other than that of an ordinary witness, ($1 a day, I believe). The doctor can be, and is, not infrequently, compelled, under penalty of a fine and imprisonment for refusal, to make a post-mortem examination at an inquest, and that too, without pay. See and re-read the excellent address by Congressman Henry in the June number of this JOURNAL, page 684. Chief Justice Gaines is there quoted as follows: "A post-mortem examination at a coroner's inquest is frequently necessary for the detection and punishment of crime. It does not seem just to impose this duty without compensation upon a learned and enlightened profession whose custom it is not to refuse the calls of charity. But they must look to the Legislature for relief. We can only declare the law as we find it; and as it now stands we think there is no provision for their compensation." Mr. Henry adds: "This broad suggestion thrown out by the learned Chief Justice has gone unheeded by the Legislature." Is the doctor not entitled to something at the hands of his government in return for all this? Surely, he is. But he has asked for his rights time and again and been answered with a snub. The Solons have always justified (?) themselves in refusing to pass an act requiring those who practice medicine to give some evidence of their qualification to do so, by the declaration that such law would be "class legislation," and for the benefit of the doctors. The stupidity of the average legislator is so dense that he can not see here the entire unselfishness of the profession; but let us put it on that ground: Suppose we grant that such bill *would* protect the qualified, the educated physician from the bastard competition which he encounters everywhere, competition for bread and butter, on the part of ignorant pretenders who have spent neither time nor money in the acquisition of knowledge and who have none,—is the doctor not entitled, under the fundamental principles upon which the government is alleged to be founded, to something,—some protection in his rights, some *quid pro quo* for the vast benefits he bestows on society and the State?

It was Abraham Lincoln, I believe, who said: "You can fool some people sometimes but you can't fool all the people all the time." Mr. Lincoln did not know Texas doctors. Mr. Lincoln meant politicians when he was talking of "fooling." He didn't know the Texas brand; the magnified lightning-bug sort. They are "slick." They can fool the doctors every time and all the time. We have more than once protested at meetings of the State Association against a repetition of the humbling process through which we go every legislative session; but there is no use in talking, it seems, and we will go on being gulled by promises before election, and snubbed after election; and the farce will be repeated without change of program indefinitely, as it has been going on to my knowledge for twenty years; the same sort fellows will be elected, and the honey-fuggling act will lose none of its sweetness.

